# Association of Launch Price and Clinical Value With Reimbursement Decisions for Anticancer Drugs in China

**DOI:** 10.34172/ijhpm.2024.8150

**Published:** 2024-04-22

**Authors:** Jing Zhou, Hao Lu, Jay Pan

**Affiliations:** ^1^HEOA Group, West China School of Public Health and West China Fourth Hospital, Sichuan University, Chengdu, China.; ^2^Institute for Healthy Cities and West China Research Center for Rural Health Development, Sichuan University, Chengdu, China.; ^3^School of Public Health, Imperial College London, London, UK.; ^4^School of Public Administration, Sichuan University, Chengdu, China.

**Keywords:** Price, Clinical Value, Reimbursement Decision, Reimbursement Lag, Anticancer Drugs, China

## Abstract

**Background:** The potential role played by launch price and clinical value in reimbursement decisions has not been sufficiently established in China. This study aimed to investigate the association of launch price and clinical value with reimbursement decisions for anticancer drugs after the implementation of reimbursement-linked price negotiation in China.

**Methods:** Anticancer drugs approved by the National Medical Products Administration (NMPA) of China from January 2017 to June 2022 were eligible for inclusion. Approval and reimbursement dates of included drug indications were retrieved from publicly available resources. We collected measures of clinical value, including survival, quality of life (QoL), and overall response rate from pivotal clinical trials and calculated treatment price at launch. Univariate and multivariate Cox proportional hazards models were employed to estimate the association between launch price, clinical value, and reimbursement decisions of anticancer drugs in China.

**Results:** The median reimbursement lag was 579 days (interquartile range [IQR]: 402–936) for 93 indications supported by randomized controlled trials and 637 days (IQR: 373–858) for 42 indications supported by single-arm clinical trials. Reimbursement was granted to 60 (65%) and 23 (55%) indications supported by randomized controlled and single-arm clinical trials, respectively. The launch price of anticancer drugs was not associated with reimbursement decisions in multivariate regression analyses. Indications supported by randomized controlled trials with higher clinical value were more likely to be reimbursed (hazard ratio [HR] for survival=1.07, 95% CI: 1.00–1.15, *P*=.037), while the overall response rate of indications supported by single-arm clinical trials was not associated with the likelihood of being reimbursed (HR=2.09, 95% CI: 0.14–32.28, *P*=.595).

**Conclusion:** The launch price of anticancer drugs may not have a significant impact on reimbursement decisions, while the implementation of reimbursement-linked price negotiation in China has prioritized anticancer drugs with higher clinical value, but only for indications supported by randomized controlled trials. Efforts are needed to prioritize indications supported by single-arm clinical trials that have higher value during the process of price negotiation.

## Background

**Key Messages**

**Implications for policy makers**

- The reimbursement lag in China following the implementation of reimbursement-linked price negotiation remains notably substantial, although significant improvements have been observed compared to the pre-implementation period.
- Indications of anticancer drugs supported by randomized controlled trials with higher clinical value were more likely to be reimbursed, while clinical value of indications supported by single-arm clinical trials was not associated with the likelihood of being reimbursed.
- China has prioritized anticancer drugs with higher clinical value and practiced value-based strategic procurement of medical insurance fund after the implementation of reimbursement-linked price negotiation, but only for indications supported by randomized controlled trials.
- Efforts to prioritize indications supported by single-arm clinical trials that have higher clinical value are needed in the process of price negotiation.

**Implications for the public**

Since 2017, China has strategically implemented reimbursement-linked drug price negotiations to address unmet clinical needs, improve the affordability of new drugs, and ensure the sustainability of the medical insurance fund through value-based strategic procurement. Our study shows that China has significantly alleviated the reimbursement lag of anticancer drugs during the post-implementation period. Additionally, the findings indicate that China has prioritized anticancer drugs with higher clinical value in the price negotiation process. However, further efforts are necessary to meet the clinical needs of patients in a more timely manner and to further prioritize high-value anticancer drugs. This study provides crucial insights into the status and associated factors of reimbursement decisions for anticancer drugs in China, which are vital determinants of patient access, thereby offering important information for the public.

Over the past decades, numerous new drugs have been approved driven by booming innovation and regulatory reform.^1-4^ New drugs have the potential to significantly improve the health outcomes of patients who typically have few treatment alternatives.^5^ However, patients will only gain benefits when these drugs enter clinical practice at affordable prices. In countries where prescription costs are covered by third-party public payers, reimbursement is a critical determinant of patient access. The role of payers, therefore, has become more prominent, and time-to-market no longer means time-to-licensing but time-to-reimbursement.^6^

 Reimbursement lag, defined as the time between market authorization and drug reimbursement, is a major obstacle to overcome in accelerating patient access to new drugs.^7^ The misalignment between drug approval and reimbursement is present in many countries, where regulatory agencies evaluate the benefits and risks of drugs for licensing, while third-party payers assess the prices of drugs to be reimbursed and their relative benefits over standard treatments.^6^ Contrasting priorities of regulators and payers can lead to discrepancies in decision-making and lags in patient access.^8^

 In China, market authorization for new drugs is granted by National Medical Products Administration (NMPA), and reimbursement decisions are made by the National Healthcare Security Administration (NHSA) (Supplementary file 1, Text S1 and Table S1). Before 2016, new drugs in China were plagued for substantial reimbursement lags following regulatory approval, and high-priced innovative drugs were rarely included in the National Reimbursement Drug List (NRDL) due to fiscal and administrative barriers.^9^ To address the issue of high prices and promote patient access, as well as ensure the sustainability of the medical insurance fund, China has been formally implementing national reimbursement-linked price negotiation annually since 2017. China has conducted six rounds of price negotiations directly with pharmaceutical companies until January 2023, resulting in the inclusion of a large number of successfully negotiated new drugs in the NRDL.^10,11^ The implementation of price negotiation in China has contributed to better patient access and affordability of new drugs.^12^

 Nevertheless, similar in many other countries, the growing number of newly approved drugs, escalating drug expenditure, and the pressure to balance budgets could always lead Chinese health authorities to be more restrictive in their decisions to reimburse high-priced drugs.^13^ To realize value-based strategic procurement of the medical insurance fund, the NHSA emphasizes the fundamental role of health technology assessment in the decision-making process during the drug price negotiations, and prioritizes ensuring timely access to drugs that are medically necessary, have favorable efficacy and safety profiles, and reasonable prices.^9^ In this context, it is expected that drug launch price would have a negative association, while clinical value would have a positive association with reimbursement decisions.

 However, the potential role of launch price and clinical value played in the reimbursement decisions has not been sufficiently established in China. Although a few studies have focused on factors associated with reimbursement decisions in China, these studies did not take into account reimbursement lag, which is a critical determinant of access and holds significance for both patients and policy-makers.^14,15^ Furthermore, while studies have investigated the reimbursement lag of drugs in Canada, Australia, South Korea, and Japan, the impact of clinical value on reimbursement decisions remains under-researched.^7,16^

 To bridge the gap in the literature, this study aimed to evaluate the association of launch price and clinical value with reimbursement decisions for anticancer drugs, taking into account the reimbursement lag in China after the implementation of national reimbursement-linked price negotiation. Evidence in this regard would help better understand the effectiveness of value-based decision-making in China’s current price negotiation process and provide policy implications for China and other countries utilizing comparable reimbursement instruments.

## Methods

###  Sample Selection

 Anticancer drugs along with their indications that were approved by the NMPA from January 2017 to June 2022 were identified from the official websites of the NMPA.^17,18^ We identified their reimbursement status from the official NRDLs, and both reimbursed and unreimbursed anticancer drugs were eligible for inclusion. For reimbursed anticancer drugs, we included the initial listed indications for drugs that successfully went through price negotiations and excluded indication extensions for drugs already listed in the NRDL. This criterion was established because the negotiation of an extension begins from the reimbursement price, and their reimbursement timelines differ from those of the initial listed indications.^19,20^ We distinguished between indications supported by randomized controlled trials and indications supported by single-arm clinical trials, given their differences in trial design and the measurement of clinical value. Ethical approval was not required for this study as human subjects were not involved.

###  Data Sources and Extraction

 As of April 4, 2023, the dates of market authorization and reimbursement dates of therapeutic indications were identified from the NMPA^18^ and the NHSA,^21^ respectively. We extracted and reviewed drug labels and review reports publicly available from the NMPA, supplemented by searching Drugdataexpy, a Chinese pharmaceutical database, if necessary.^18,22^ We identified the pivotal clinical trial of each included indication in the section of “pivotal studies” in the review report or referring to the drug label when the review report was not available. When multiple trials existed, the trial with data available that best matched the indication, targeted the Chinese or Asian population, or had the best clinical outcome was selected sequentially.^23^

 To assess the clinical value of therapeutic indications supported by randomized controlled trials, we extracted information on overall survival (OS), progression-free survival (PFS), and quality of life (QoL) of pivotal studies from peer-reviewed publications.^24-26^ All extracted information should be available at the time of price negotiation. The median OS, or PFS when OS was unavailable, in the experimental and control arms, were extracted, and the absolute difference between the two arms as added survival benefits was calculated for each indication.^27,28^ In cases indications had more than one clinical trial eligible for selection based on clinical outcomes, we selected the one that had the best OS gain in months. The QoL for the experimental group, as compared to the control group, was categorized into three categories, namely, improvement, no difference, and reduced or unavailable, after reviewing the relevant contents in the publication for the pivotal study.^24^ For indications supported by single-arm clinical trials, we retrieved objective response rates (ORRs) from pivotal clinical trials as an indicator of clinical value.

 We retrieved the launch price of anticancer drugs from Drugdataexpy.^29^ The treatment price for each therapeutic indication over an expected treatment duration were estimated based on dosing information from drug labels. This approach accounts for differences in the duration of treatment across anticancer drugs and therapeutic indications.^27,30^ The median treatment duration for each indication was collected from the pivotal trial.^30^ For therapeutic indications for which dosages depended on body surface area or weight, we assumed a patient weighing 60 kg with a body surface area of 1.6 m^2^ in consistent with the NHSA requirements for dossiers of drugs to be negotiated.

 We also collected data on whether the anticancer drug was domestically developed, whether the therapeutic indication was approved through priority review or conditional approval, as well as the cancer site, the drug type, the line of therapy, the trial characteristics, and the administration route as control variables.^28^ An overview of all variables can be found in the supplementary (Table S2).

###  Statistical Analysis

 Numeric variables were presented as medians and interquartile ranges (IQRs), whereas categorical variables were represented as frequency and percentage. The cumulative risk curves were used to illustrate the time between market authorization and reimbursement for included indications stratified by trial design. We used Cox proportional hazards model of survival analysis to estimate the impact of launch price and clinical value on reimbursement decisions for indications supported by randomized controlled and single-arm clinical trials, respectively. Survival analysis is concerned primarily with analyzing “time” to the “occurrence of events.” In our study, time was calculated in days from market authorization to reimbursement and event refers to the incorporation of indications for anticancer drugs in the NRDL (ie, reimbursement). For indications that were not listed in the NRDL by 2023, we estimated the time from market authorization to December 31, 2023, as the NRDL will not be renewed until 2024. We analyzed the unadjusted and adjusted hazard ratios (HRs) of the events, reflecting the instantaneous likelihood that indications entering the NRDL, using univariate and multivariate Cox proportional hazard models, respectively. In the multivariate Cox proportional hazards model, potential confounding factors were adjusted by using a backward procedure with a removal criterion of* P* value greater than.05.^31^ We performed the above analyses separately for indications supported by randomized controlled and single-arm clinical trials.

###  Sensitivity Analysis

 We conducted three different sensitivity analyses to confirm the robustness of our findings. First, we used structural accelerated failure time models with various statistical distributions (eg, Weibull, extreme) to assess the effect of launch price and clinical value on reimbursement decisions.^32^ Second, the logistic regression modeling the likelihood of entering the NRDL was employed. Third, survival as the measure of the clinical value of indications supported by randomized controlled trials was replaced by PFS, with OS as a supplementary where PFS data was unavailable.

 All data were collected using a pre-designed Excel file and were imported into R (version 4.1.0) for statistical analysis. The ggplot2 (version 3.3.5) was used for visualization. All statistical tests were two-sided, and a two-tailed *P* value <.05 was considered to be statistically significant.

## Results

**Figure 1:**
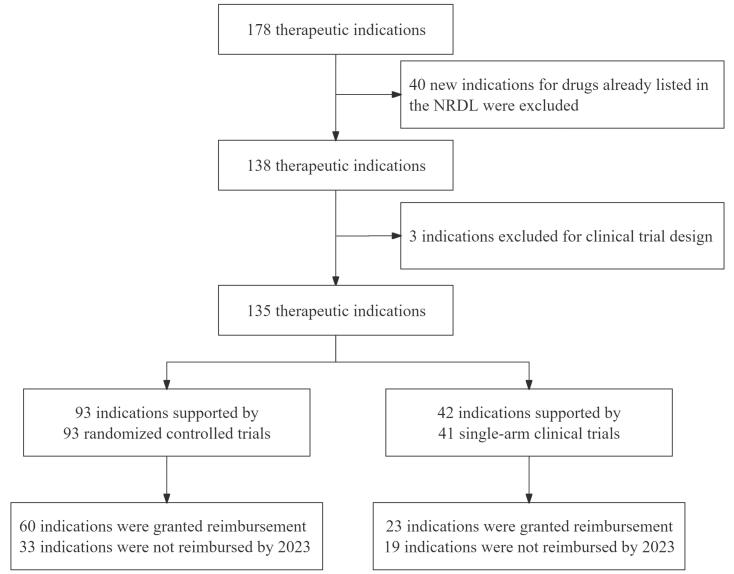
Flowchart of Sample Selection. Notes: Out of 93 indications supported by randomized controlled trials, 20 had more than one pivotal randomized controlled trial, and out of 42 indications supported by single-arm clinical trials, 7 had more than one pivotal single-arm clinical trial. Therefore, we sequentially selected the trial with available data that best matched the indication, targeted the Chinese or Asian population, or had the best clinical outcome. Abbreviation: NRDL, National Reimbursement Drug List.

**Figure 2:**
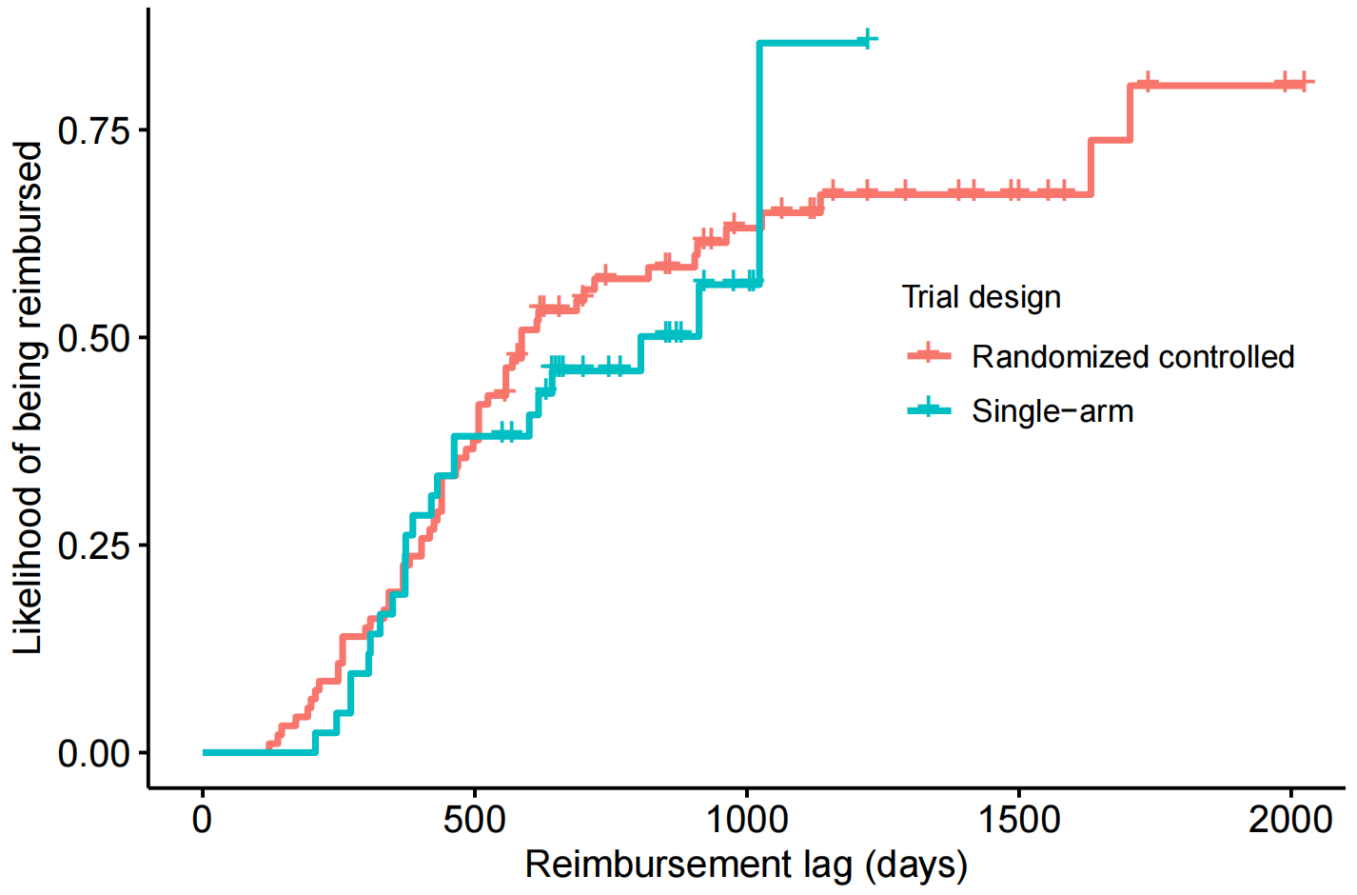
The Likelihood of Being Reimbursed Over Time for Included Indications Stratified by Trial Design in China. Notes: The cumulative risk curves illustrate the likelihood of being reimbursed over time (reimbursement lag) for included indications supported by both randomized controlled (n = 93) and single-arm (n = 42) clinical trials. Reimbursement lag was calculated as the time from market authorization to reimbursement.

 From January 2017 to June 2022, 178 therapeutic indications for 95 anticancer drugs were approved by the NMPA. After excluding 40 indication extensions for drugs already listed in the NRDL and three indications whose arms included the study drug simultaneously, we included 93 indications supported by randomized controlled trials and 42 indications supported by single-arm clinical trials in our study (Figure 1, Tables S3-S4). The median reimbursement lag for all 135 included indications was 586 days (IQR: 394–911), with indications supported by randomized controlled trials having a median lag of 579 days (IQR: 402–936), and those supported by single-arm clinical trials having a median lag of 637 days (IQR 373–858) (Figure 2 and Tables 1-2). By 2023, reimbursement was granted to 60 (65%) indications supported by randomized controlled trials and 23 (55%) indications supported by single-arm clinical trials, while 33 (35%) and 19 (45%) indications, respectively, were not reimbursed (Tables 1-2).

**Table 1:** Characteristics of Included Therapeutic Indications Supported by Randomized Controlled Trials in China

**Characteristics**	**Total (n = 93)**	**Reimbursed (n = 60)**	**Not Reimbursed (n = 33)**
**Categorical Variables, No. (%)**			
Drug type			
Small targeted molecule	40 (43)	39 (65)	1 (3)
Targeted monoclonal antibody	12 (13)	9 (15)	3 (9)
Immunotherapy	27 (29)	3 (5)	24 (73)
Endocrine therapy and chemotherapy	14 (15)	9 (15)	5 (15)
Cancer site			
Blood	16 (17)	12 (20)	4 (12)
Lung	21 (23)	10 (17)	11 (33)
Breast	13 (14)	13 (22)	0 (0)
Gastric	10 (11)	3 (5)	7 (21)
Other	33 (35)	22 (37)	11 (33)
Origin country			
Imported	75 (81)	44 (73)	31 (94)
Domestic	18 (19)	16 (27)	2 (6)
Priority review			
No	32 (34)	16 (27)	16 (48)
Yes	61 (66)	44 (73)	17 (52)
Administration route			
Oral	45 (48)	43 (72)	2 (6)
Intravenous	48 (52)	17 (28)	31 (94)
First-line therapy			
No	51 (55)	37 (62)	14 (42)
Yes	42 (45)	23 (38)	19 (58)
Reference drug			
Placebo	24 (26)	19 (32)	5 (15)
Positive	69 (74)	41 (68)	28 (85)
Blind			
No	51 (55)	31 (52)	20 (61)
Yes	42 (45)	29 (48)	13 (39)
Quality of life			
No difference	30 (32)	22 (37)	8 (24)
Improvement	26 (28)	17 (28)	9 (27)
Reduction or not available	37 (40)	21 (35)	16 (48)
**Continuous Variables, Median (IQR)**			
Reimbursement lag (n = 95)	579 (402–936)	439 (320–586)	978 (741–1419)
Launch price^a^ (n = 92)	CNY 312 939 (139 035–572 000) US$ 46 450 (20 637–84 904)	CNY 353 243 (134 696–628 290) US$ 52 433 (19 991–93 260)	CNY 269 722 (169 078–394 196) US$ 40 035 (25 097–58 512)
Survival in either OS or PFS^b^ (n = 77)	4.20 (2.50–8.20)	6.40 (2.75–10.15)	3.60 (2.40–5.10)

Abbreviations: OS, overall survival; PFS, progression-free survival; IQR, interquartile range. ^a^ Information was not available for 3 indications. ^b^ Information was not available for 18 indications. Reimbursement lag for unreimbursed drugs was calculated from the date of their regulatory approval to December 31, 2023. It is important to note that this lag may extend beyond the presented data, as these drugs may remain unreimbursed after the cutoff date.

**Table 2:** Characteristics of Included Therapeutic Indications Supported by Single-Arm Clinical Trials in China

**Characteristics **	**Total (n = 42)**	**Reimbursed (n = 23)**	**Not Reimbursed (n = 19)**
**Categorical Variables, No. (%)**			
Drug type			
Small targeted molecule	25 (60)	14 (60)	11 (58)
Targeted monoclonal antibody	17 (40)	9 (40)	8 (42)
Cancer sites			
Hematological	21 (50)	13 (57)	8 (42)
Non-hematological	21 (50)	10 (43)	11 (58)
Origin country			
Imported	18 (40)	7 (30)	11 (58)
Domestic	24 (60)	16 (70)	8 (42)
Conditional approval			
No	8 (19)	3 (13)	5 (26)
Yes	34 (81)	20 (87)	14 (74)
Administration route			
Oral	21 (50)	13 (57)	8 (42)
Intravenous	21 (50)	10 (43)	11 (58)
First-line therapy			
No	34 (81)	18 (78)	16 (84)
Yes	8 (19)	5 (22)	3 (16)
**Continuous Variables, Median (IQR)**			
Reimbursement lag (n = 42)	637 (373–858)	386 (317–609)	767 (652–901)
Launch price^a^ (n = 37)	CNY 308 000 (188 112–487 500) US$ 45 718 (27 922–72 362)	CNY 308 000 (185 531–463 125) US$ 45 718 (27 539–68 743)	CNY 301 730 (215 280–665 470) US$ 44 787 (31 955–98 778)
ORR^b^ (n = 39)	0.60 (0.39–0.78)	0.70 (0.43–0.77)	0.51 (0.39–0.79)

Abbreviations: ORR, objective response rate; IQR, interquartile range. ^a^ Information was not available for 5 indications. ^b^ Information was not available for 3 indications. Reimbursement lag for unreimbursed drugs was calculated from the date of their regulatory approval to December 31, 2023. It is important to note that this lag may extend beyond the presented data, as these drugs may remain unreimbursed after the cutoff date.

 The launch price over expected treatment durations was CNY 312 939 (IQR: 139 035–572 000) [US$ 46 450 (IQR: 20 637–84 904)] for indications supported by randomized controlled trials and CNY 308 000 (IQR: 188 112–487 500) [US$ 45 718 (IQR: 27 922–72 362)] for indications supported by single-arm clinical trials (Tables 1-2). The clinical value of indications supported by randomized controlled trials was measured by survival in either OS or PFS, and QoL, and the former had a median value of 4.20 (IQR: 2.50–8.20) months. As for QoL, out of the 93 indications included in the study, 30 had no difference, 26 had an improvement, and 37 had a reduction or unavailable data in QoL compared to their reference drugs. The median ORR, used as the clinical value indicator for indications supported by single-arm clinical trials, was 0.60 (IQR: 0.39–0.78).

 For indications supported by randomized controlled trials, the univariate analysis (Table 3) revealed a positive association between launch price and the likelihood of therapeutic indications entering the NRDL (HR = 1.00, 95% CI: 1.00–1.00, *P* = .007). While each additional life-month gained in either OS or PFS was associated with a 9% increase in the likelihood of being reimbursed (HR = 1.09, 95% CI: 1.04–1.15, *P* < .001), indications showing improvement (HR = 1.27, 95% CI: 0.67 - 2.41, *P* = .471) or no ‎difference (HR = 1.77, 95% CI: 0.97–3.22, *P* = .064) in QoL exhibited a ‎higher likelihood of being listed in the NRDL compared to those showing reduction or ‎with unavailable data, although the differences were not statistically significant‎. Small targeted molecules, domestically developed, approved through priority review, and orally administrated were all associated with higher likelihood of being reimbursed. On the other hand, cancer site, first-line therapy and trial characteristics (reference drug and the blind method) did not significantly impact reimbursement of therapeutic indications.

**Table 3:** Factors Associated With the Likelihood of Being Reimbursed for Therapeutic Indications Supported by Randomized Controlled Trials in China

**Characteristics **	**Unadjusted HR**	**95% CI**	***P*** ** Value**	**Adjusted HR**	**95% CI**	***P*** ** Value**
Treatment price	1.00	1.00–1.00	.007	1.00	1.00–1.00	.588
Survival in either OS or PFS	1.09	1.04–1.15	<.001	1.07	1.00–1.15	.037
QoL						
Reduction or not available	1			1		
No difference	‎1.77‎	‎0.97–3.22‎	‎0.064‎	‎2.75‎	‎1.23–6.14‎	‎.013‎
Improvement	‎1.27‎	‎0.67–2.41‎	‎0.471‎	‎2.66‎	‎1.14–6.21‎	‎.023‎
Cancer site						
Blood	1			NA	NA	NA
Lung	0.45	0.19–1.06	.066	NA	NA	NA
Breast	1.22	0.55–2.69	.621	NA	NA	NA
Gastric	0.29	0.08–1.03	.055			
Other	0.79	0.39–1.58	.505	NA	NA	NA
Drug type						
Small targeted molecule	1			1		
Targeted monoclonal antibody	0.65	0.33–1.29	.222	1.81	0.31–10.64	.509
Immunotherapy	0.03	0.01–0.11	<.001	0.10	0.01–0.72	.022
Endocrine therapy and chemotherapy	0.34	0.16–0.73	.006	1.15	0.41–3.26	.789
Origin country						
Imported	1			1		
Domestic	3.64	2.00–6.64	<.001	5.13	2.36–11.14	<.001
Priority review						
No	1			NA	NA	NA
Yes	1.87	1.05–3.31	.032	NA	NA	NA
Administration route						
Oral	1			1		
Intravenous	0.15	0.08–0.28	<.001	0.31	0.07–1.30	.109
First-line therapy						
No	1			NA	NA	NA
Yes	0.55	0.33–0.92	.024	NA	NA	NA
Reference drug						
Placebo	1			NA	NA	NA
Positive	0.58	0.33–1.01	.054	NA	NA	NA
Blind						
No				NA	NA	NA
Yes	1.34	0.81–2.24	.257	NA	NA	NA

Abbreviations: QoL, quality of life; HR, hazard ratio; CI, confidence interval; OS, overall survival; PFS, progression-free survival; NA, not applicable.

 As a result of the backward elimination process, launch price, survival in either OS or PFS, QoL, drug type, origin country, and administration route were included in the multivariate regression analysis (Table 3). The impact of clinical value on the likelihood of reimbursement remained highly consistent with the univariate analysis, indicating that higher clinical value (survival in either OS or PFS, and QoL) increased the likelihood of being listed in the NRDL. However, the impact of launch price on reimbursement decisions became insignificant, with the estimates largely unchanged. Additionally, the hazard ratio for domestically developed indications was 5.13 times higher than foreign-developed indications (HR = 5.13, 95% CI: 2.36–11.14, *P* = .0.001), implying a faster reimbursement for home-grown indications. Immunotherapy had a significantly lower likelihood of being listed in the NRDL than small targeted molecules, with a 90% decrease in hazard ratio (HR = 0.10, 95% CI: 0.01–0.72, *P* = .022).

 For indications supported by single-arm clinical trials, both univariate and multivariate regression analysis indicated that launch price and ORR did not have a significant impact on reimbursement decisions (Table 4). While domestically developed indications seemed more likely to be reimbursed in the univariate analysis (HR = 3.49, 95% CI: 1.27–9.63, *P* = .016), this effect did not reach statistical significance in the multivariate regression analysis (HR = 3.45, 95% CI: 0.97–12.35, *P* = .057). Drug type, cancer site, administrated route, line of therapy and conditional approval did not affect the likelihood of being listed in the NRDL.

**Table 4:** Factors Associated With the Likelihood of Being Reimbursed for Therapeutic Indications Supported by Single-Arm Clinical Trials in China

**Characteristics **	**Unadjusted HR**	**95% CI**	***P*** ** Value**	**Adjusted HR**	**95% CI**	***P*** ** Value**
Treatment price	1.00	1.00–1.00	.139	1.00	1.00–1.00	.426
ORR	1.57	0.25–10.04	.631	2.09	0.14–32.28	.595
Drug type						
Small targeted molecule	1.00			1.00		
Targeted monoclonal antibody	0.72	0.31–1.69	.454	0.87	0.04–21.08	.933
Cancer sites						
Hematological	1.00			1.00		
Non-hematological	1.06	0.45–2.50	.895	0.95	0.29–3.08	.932
Origin country						
Imported	1.00			1.00		
Domestic	3.49	1.27–9.63	.016	3.45	0.97–12.35	.057
Conditional approval						
No	1.00			1.00		
Yes	2.74	0.79–9.58	.081	2.67	0.25–28.77	.417
Administration route						
Oral	1.00			1.00		
Intravenous	0.41	0.17–1.01	.053	0.49	0.02–13.03	.667
First-line therapy						
No	1.00			1.00		
Yes	1.47	0.53–4.03	.450	1.11	0.34–3.67	.860

Abbreviations: ORR, objective response rate; HR, hazard ratio; CI, confidence interval.

###  Sensitivity Analysis

 The results of the sensitivity analyses using alternative regression models remained highly consistent with the main analyses. Based on the structural accelerated failure time models and the logistic models, the clinical value of indications supported by randomized controlled trials was positively associated with the likelihood of being listed in the NRDL, while the association was not statistically significant for indications supported by single-arm clinical trials. The launch price for indications supported by both randomized controlled and single-arm clinical trials remained insignificant in predicting reimbursement decisions in multivariate regression analyses. In the third sensitivity analysis, where the survival measure was replaced with PFS and OS as supplementary measure for indications supported by randomized controlled trials, no significant changes in the adjusted hazard ratio were observed (HR = 1.08, 95% CI: 1.02–1.15, *P* = .009).

## Discussion

 To the best of our knowledge, this is the first study to investigate the impact of launch price and clinical value on reimbursement decisions, considering the reimbursement lag of anticancer drugs in China. The study findings demonstrated that indications supported by randomized controlled trials with higher clinical value were more likely to be reimbursed, while this association was not observed for single-arm clinical trials. Furthermore, the launch price may not have an impact on the likelihood of reimbursement for indications supported by either randomized controlled or single-arm clinical trials.

 Reimbursement is essential for promoting equitable access to medicines, it reduces the financial burden on patients, improves medication adherence, and ultimately leads to better health outcomes.^33^ We found that the median time from market authorization to reimbursement of our study sample was 586 days, which was much shorter than four to nine years observed in the pre-implementation period of reimbursement-linked price negotiation.^34^ Nevertheless, the reimbursement lag in China during the post-implementation period was still considerably longer than that in many other countries. In European countries, the median reimbursement lag was 469 days, with 28 out of 37 countries experiencing reimbursement lags of less than 600 days.^35^ Additionally, a reimbursement lag of 258 days was reported in Canada, 137 days in Australia, 58 days in Japan, and 368 days in South Korea.^7^ This comparison underscores the ongoing need for efforts to improve reimbursement efficiency and reduce lag times for anticancer drugs in China.

 Policy-makers and payers are increasingly confronted with the high price tags of new drugs, raising concerns about the financial sustainability of publicly funded healthcare systems.^13^ Studies of reimbursement decisions consistently find that economic considerations have exerted major influences, with countries becoming more cautious and conservative in granting reimbursement decisions for expensive drugs that may not provide sufficient value for money.^13,36-38^ These findings, which include empirical evidence from Poland and South Korea showing that drug prices of anticancer drugs were negatively associated with the likelihood of being reimbursed,^37,38^ differ from the findings in our study. Surprisingly, we found that the launch price of anticancer drugs in China may not have an impact on the likelihood of being reimbursed. This may be explained by the fact that China has placed a high priority on meeting patients’ clinical needs and addressing therapeutic gaps in the NRDL, since many new anticancer drugs were not authorized nor listed in the NRDL before 2016.^2,9^ In addition, with the implementation of price negotiation, high-priced anticancer drugs were likely subject to substantial price reductions, which may offset the negative influence of high launch prices on reimbursement decisions.^12^

 In our study, we assessed the clinical value of indications supported by randomized controlled trials using survival in either OS or PFS, along with QoL. In China, OS and PFS are considered primary clinical endpoints, with OS generally preferred when both are available.^39^ QoL, as an important subjective measure, is also valued in China.^23,39^ We found that the clinical value of indications supported by randomized controlled trials was positively associated with the likelihood of being listed in the NRDL. This suggests that, during the implementation process of price negotiation, China has prioritized treatments demonstrating comparative clinical benefits as part of its value-based strategic procurement and policy goals.

 This finding is in line with other studies revealing that decision-makers are more likely to grant positive reimbursement decisions to drugs with higher clinical benefits,^36,37,40^ and in contrast with studies in Canada, Belgium, Estonia, Scotland, Slovenia, and Sweden, which have shown no or low correlation between clinical value and time to reimbursement or access.^41,42^ The magnitude of clinical value of anticancer drugs varies widely, and an appreciable proportion of drugs offer no to little added benefits over existing drugs.^43,44^ Therefore, alleviating reimbursement lag and increasing the likelihood of reimbursement for anticancer drugs with greater clinical value, rather than those with low clinical value, has great potential to maximize health outcomes, especially in the context of limited resources.^33,45-47^

 Existing evidence on the association between clinical value for indications supported by single-arm clinical trials and reimbursement decisions is currently limited. In our study, we utilized ORR to assess the clinical value of indications supported by single-arm clinical trials, as it is the most commonly used primary endpoint in such trials.^48^ Unfortunately, we did not find evidence supporting a higher ORR of anticancer drugs being associated with a higher probability of reimbursement in China. To facilitate access to new drugs, single-arm clinical trials are increasingly considered sufficient for drug approvals and reimbursement.^49^ Indications supported by single-arm clinical trials in China were mostly approved through conditional approval, a specialized process for approving health technologies that treat serious, life-threatening diseases for which there are no effective treatments. The lack of association between clinical value and reimbursement decisions highlights the importance for health authorities to prioritize indications supported by single-arm clinical trials with higher clinical benefits, particularly when these treatments are urgently needed. However, it should be noted that drug approvals based on single-arm clinical trials may rely on false positive, exaggerated, and/or ‘fragile’ findings.^50^ Therefore, routine monitoring of the clinical value of these drugs and timely updates of clinical outcomes from confirmatory trials are necessary.^43,50^ Additionally, the use of randomized controlled trials as supporting evidence for pricing and reimbursement whenever possible should be further encouraged.

###  Study Limitations

 This study has several limitations. First, while China has been increasing transparency in the decision-making process of price negotiation, the availability of relevant data has been limited to varying degrees, particularly in previous years. Therefore, our study relied on data obtained from other sources, including publications and publicly available databases, which may not fully represent the information reviewed by health authorities. In addition, all measures of clinical value in our study originated from pivotal clinical trials, which cannot fully represent the effectiveness of drugs in real-world settings due to uncertainty in clinical evidence, although they are crucial for informing pricing and reimbursement decisions. Second, in cases where OS data in median times were not available, we used added survival in PFS as a surrogate. Additionally, one category of QoL was labeled as “reduced or unavailable” due to the low rate of QoL reporting. Evidence showed that the reporting of QoL in clinical trials was associated with positive trial outcomes, while harm was often under-reported.^51-53^

 Third, we only included measures of clinical value in our analysis. As drug evaluation evolves toward comprehensive value assessment, subsequent rounds of price negotiation in China have incorporated measures reflecting other aspects of value, such as equity and innovation. Future studies could build on this research by incorporating additional elements of value. Fourth, we excluded supplementary indications for anticancer drugs that were already listed in the NRDL due to differences in pricing mechanisms and reimbursement timelines compared to the initial listed indications.^19,20^ Finally, our study did not identify factors relevant to price negotiation that could enhance the association between clinical value and the likelihood of being reimbursed, future studies are encouraged to investigate this aspect.

## Conclusion

 The launch price of anticancer drugs may not have an impact on the reimbursement decisions of anticancer drugs in China. A positive association between clinical value and the likelihood of being reimbursed was observed for indications supported by randomized controlled trials, while the ORR of indications supported by single-arm clinical trials was not associated with the likelihood of being listed in the NRDL. These findings suggest that China has prioritized anticancer drugs with higher clinical value and practiced value-based strategic procurement of medical insurance fund after the implementation of national reimbursement-linked price negotiation, but only for indications supported by randomized controlled trials. Efforts to prioritize indications supported by single-arm clinical trials that have higher clinical value are needed in the process of price negotiation. The evidence from China could enlighten other countries that have adopted comparable reimbursement instruments, though the dynamics of reimbursement varies in different countries.

## Acknowledgements

 The authors would like to thank TianJiao Lan from West China School of Public Health and West China Fourth Hospital of Sichuan University for his helpful suggestions on methodology.

## Ethical issues

 Ethical approval was not required for this study as human subjects were not involved.

## Competing interests

 Authors declare that they have no competing interests.

## Funding

 This work was supported by National Natural Science Foundation of China (Grant No. 72074163, and 72374149), and China Center for South Asian Studies, Sichuan University. The funders had no influence on study design, data analysis, preparation of the manuscript, or decision to publish.

## Supplementary files

Supplementary file 1 contains Text S1 and Tables S1-S4.

## References

[1] Zhang Y, Wagner AK, Guan X (2023). Newly approved cancer drugs in China - innovation and clinical benefit. Nat Rev Clin Oncol.

[2] Liu Y, Zhang N, Xie C (2022). Evolution of drug regulations and regulatory innovation for anticancer drugs in China. Acta Pharm Sin B.

[3] Kesselheim AS, Wang B, Franklin JM, Darrow JJ (2015). Trends in utilization of FDA expedited drug development and approval programs, 1987-2014: cohort study. BMJ.

[4] Zhang Y, Naci H, Wagner AK (2022). Overall survival benefits of cancer drugs approved in China from 2005 to 2020. JAMA Netw Open.

[5] Zhu X, Liu B. Launch delay of new drugs in China and effect on patients’ health. Clin Ther 2020;42(9):1750-1761.e7. 10.1016/j.clinthera.2020.06.023. 32798058

[6] Eichler HG, Bloechl-Daum B, Abadie E, Barnett D, König F, Pearson S (2010). Relative efficacy of drugs: an emerging issue between regulatory agencies and third-party payers. Nat Rev Drug Discov.

[7] Shih YR, Liao KH, Chen YH, Lin FJ, Hsiao FY (2020). Reimbursement lag of new drugs under Taiwan’s national health insurance system compared with United Kingdom, Canada, Australia, Japan, and South Korea. Clin Transl Sci.

[8] Zannad F, de Los Angeles Alonso Garcia M, Borer JS (2017). Role of payers in the development of cardiovascular therapeutics: misalignment between approval and reimbursement. J Am Coll Cardiol.

[9] Liu GG, Wu J, He X, Jiang Y (2022). Policy updates on access to and affordability of innovative medicines in China. Value Health Reg Issues.

[10] Li H, Liu GG, Wu J, Wu JH, Dong CH, Hu SL (2018). Recent pricing negotiations on innovative medicines pilot in China: experiences, implications, and suggestions. Value Health Reg Issues.

[11] Zhou J, Lan T, Lu H, Pan J (2024). Price negotiation and pricing of anticancer drugs in China: an observational study. PLoS Med.

[12] Zhang Y, Wushouer H, Han S (2021). The impacts of government reimbursement negotiation on targeted anticancer medication price, volume and spending in China. BMJ Glob Health.

[13] Vogler S, Paris V, Panteli D. Ensuring Access to Medicines: How to Redesign Pricing, Reimbursement and Procurement? WHO Regional Office for Europe; 2018. 30272895

[14] Wen J, Li M, Jiang Y (2023). Cost effectiveness of innovative anti-cancer drugs and reimbursement decisions in China. Health Policy Technol.

[15] Ling K, Qin H, Feng Y, Che H, Ding J, Li W (2022). Correlation between clinical trial endpoints of marketed cancer drugs and reimbursement decisions in China. Front Public Health.

[16] Salek S, Lussier Hoskyn S, Johns JR, Allen N, Sehgal C (2019). Factors influencing delays in patient access to new medicines in Canada: a retrospective study of reimbursement processes in public drug plans. Front Pharmacol.

[17] National Medical Products Administration. 2021 Drug Review Report. 2022. https://www.nmpa.gov.cn/xxgk/fgwj/gzwj/gzwjyp/20220601110541120.html. Accessed March 24, 2023.

[18] Center for Drug Evaluation of NMPA. Information on Listed Drugs. https://www.cde.org.cn/main/xxgk/listpage/b40868b5e21c038a6aa8b4319d21b07d.

[19] Mills M, Michaeli D, Miracolo A, Kanavos P (2023). Launch sequencing of pharmaceuticals with multiple therapeutic indications: evidence from seven countries. BMC Health Serv Res.

[20] Michaeli DT, Michaeli T (2023). Cancer drug prices in the United States: efficacy, innovation, clinical trial evidence, and epidemiology. Value Health.

[21] National Healthcare Security Administration. Policy and Regulation. http://www.nhsa.gov.cn/col/col104/index.html. Accessed April 4, 2023.

[22] DRUGDATAEXPY. Rational Administration. https://data.yaozh.com/. Accessed March 24, 2023.

[23] Vokinger KN, Hwang TJ, Grischott T (2020). Prices and clinical benefit of cancer drugs in the USA and Europe: a cost-benefit analysis. Lancet Oncol.

[24] Salas-Vega S, Iliopoulos O, Mossialos E (2017). Assessment of overall survival, quality of life, and safety benefits associated with new cancer medicines. JAMA Oncol.

[25] Zhang Y, Wei Y, Li H (2022). Prices and clinical benefit of national price-negotiated anticancer medicines in China. Pharmacoeconomics.

[26] Kovic B, Jin X, Kennedy SA (2018). Evaluating progression-free survival as a surrogate outcome for health-related quality of life in oncology: a systematic review and quantitative analysis. JAMA Intern Med.

[27] Howard DH, Bach PB, Berndt ER, Conti RM (2015). Pricing in the market for anticancer drugs. J Econ Perspect.

[28] Lauenroth VD, Kesselheim AS, Sarpatwari A, Stern AD (2020). Lessons from the impact of price regulation on the pricing of anticancer drugs in Germany. Health Aff (Millwood).

[29] DRUGDATAEXPY. Information of Drug Winning Bid. 2022. https://db.yaozh.com/yaopinzhongbiao.

[30] Salas-Vega S, Shearer E, Mossialos E (2020). Relationship between costs and clinical benefits of new cancer medicines in Australia, France, the UK, and the US. Soc Sci Med.

[31] Lunghi C, Moisan J, Grégoire JP, Guénette L (2017). The association between depression and medication nonpersistence in new users of antidiabetic drugs. Value Health.

[32] Hernán MA, Cole SR, Margolick J, Cohen M, Robins JM (2005). Structural accelerated failure time models for survival analysis in studies with time-varying treatments. Pharmacoepidemiol Drug Saf.

[33] Leinwand B, Sollano J, Doherty JP (2019). PCN402 the clinical and economic consequences of delays in reimbursement for select novel cancer therapeutics in Canada, Italy, and Australia. Value in Health.

[34] Technology Development Center of Chinese Pharmaceutical Association. Blue Book on the Progress and Achievements of China’s Medical Insurance Drug Management Reform. 2021.

[35] IQVIA. EFPIA Patients W.A.I.T. Indicator 2022 Survey. https://www.efpia.eu/media/s4qf1eqo/efpia_patient_wait_indicator_final_report.pdf.

[36] Drummond M (2013). Twenty years of using economic evaluations for drug reimbursement decisions: what has been achieved?. J Health Polit Policy Law.

[37] Gong JR, Lee D, Lim KM, Bae S (2020). Are recently evaluated drugs more likely to receive positive reimbursement recommendations in South Korea? 11-year experience of the South Korean positive list system. Clin Ther.

[38] Malinowski KP, Kawalec P, Trąbka W (2016). Impact of patient outcomes and cost aspects on reimbursement recommendations in Poland in 2012-2014. Health Policy.

[39] China National Health Development Research Center. Notice of the National Center for Comprehensive Evaluation of Drugs and Health Technology on the Release of Technical Guidelines for Clinical Comprehensive Evaluation of Cardiovascular Diseases, Anticancer, and Pediatric Drugs. http://www.nhei.cn/nhei/znfb/202206/c01d87a290664b01bf42a9dad769d69f.shtml.

[40] Kim ES, Kim JA, Lee EK (2017). National reimbursement listing determinants of new cancer drugs: a retrospective analysis of 58 cancer treatment appraisals in 2007-2016 in South Korea. Expert Rev Pharmacoecon Outcomes Res.

[41] Thomson S, Everest L, Witzke N (2022). Examining the association between oncology drug clinical benefit and the time to public reimbursement. Cancer Med.

[42] Ferrario A (2018). Time to entry for new cancer medicines: from European Union-wide marketing authorization to patient access in Belgium, Estonia, Scotland, and Sweden. Value Health.

[43] Wieseler B, McGauran N, Kaiser T (2019). New drugs: where did we go wrong and what can we do better?. BMJ.

[44] Michaeli DT, Michaeli T (2022). Overall survival, progression-free survival, and tumor response benefit supporting initial US food and drug administration approval and indication extension of new cancer drugs, 2003-2021. J Clin Oncol.

[45] Janzic U, Knez L, Janzic A, Cufer T (2019). Time to access to novel anticancer drugs and the correlation with ESMO-Magnitude of Clinical Benefit Scale in Slovenia. Expert Rev Pharmacoecon Outcomes Res.

[46] Jommi C, Armeni P, Costa F, Bertolani A, Otto M (2020). Implementation of value-based pricing for medicines. Clin Ther.

[47] Sussex J, Towse A, Devlin N (2013). Operationalizing value-based pricing of medicines: a taxonomy of approaches. Pharmacoeconomics.

[48] Huang Y, Xiong W, Zhao J, Li W, Ma L, Wu H (2023). Early phase clinical trial played a critical role in the Food and Drug Administration-approved indications for targeted anticancer drugs: a cross-sectional study from 2012 to 2021. J Clin Epidemiol.

[49] Hilal T, Gonzalez-Velez M, Prasad V (2020). Limitations in clinical trials leading to anticancer drug approvals by the US Food and Drug Administration. JAMA Intern Med.

[50] Schnog JB, Samson MJ, Gans ROB, Duits AJ (2021). An urgent call to raise the bar in oncology. Br J Cancer.

[51] Seruga B, Templeton AJ, Badillo FE, Ocana A, Amir E, Tannock IF (2016). Under-reporting of harm in clinical trials. Lancet Oncol.

[52] Marandino L, La Salvia A, Sonetto C (2018). Deficiencies in health-related quality-of-life assessment and reporting: a systematic review of oncology randomized phase III trials published between 2012 and 2016. Ann Oncol.

[53] Saleh RR, Meti N, Ribnikar D (2020). Associations between safety, tolerability, and toxicity and the reporting of health-related quality of life in phase III randomized trials in common solid tumors. Cancer Med.

