# Supplementary material for: Association of Launch Price and Clinical Value With Reimbursement Decisions for Anticancer Drugs in China
Source: Int J Health Policy Manag. 2024 Apr 22;13:8150. doi: 10.34172/ijhpm.2024.8150 (PMC11270615; doi:10.34172/ijhpm.2024.8150)
Supplement: Supplementary file 1 — contains Text S1 and Tables S1-S4. [file ijhpm-13-8150-s001.pdf]

Article title: Association of Launch Price and Clinical Value With Reimbursement Decisions for Anticancer Drugs in China

Journal name: International Journal of Health Policy and Management (IJHPM)

Authors' information: Jing Zhou,[1,2] Hao Lu, [3] Jay Pan[1,4*]

[1]HEOA Group, West China School of Public Health and West China Fourth Hospital, Sichuan University, Chengdu, China.

[2]Institute for Healthy Cities and West China Research Center for Rural Health Development, Sichuan University, Chengdu, China.

[3]School of Public Health, Imperial College London, London, UK.

[4]School of Public Administration, Sichuan University, Chengdu, China.

***Correspondence to:** Jay Pan; Email: panjie.jay@scu.edu.cn

Citation: Zhou J, Lu H, Pan J. Association of launch price and clinical value with reimbursement decisions for anticancer drugs in China. Int J Health Policy Manag. 2024;13:8150. doi:10.34172/ijhpm.2024.8150

Supplementary file 1

Text S1. Price negotiation in China

In China, the Center for Drug Evaluation, National Medical Products Administration (CDE, NMPA), is responsible for drug approval, and the National Healthcare Security Administration (NHSA) is responsible for price negotiation and reimbursement decisions. To address the issue of high prices and promote patient access, as well as ensure the sustainability of the medical insurance fund, China formally launched the national reimbursement-linked price negotiation in 2017. Since then, China has conducted six rounds of price negotiations directly with pharmaceutical companies until January 2023, resulting in the inclusion of a large number of successfully negotiated new drugs in the NRDL (Table S1).

The process of price negotiation over the years is generally similar and can be roughly divided into the preparation stage, application stage, expert review stage, negotiation stage, and results announcement stage. [1,2] Taking 2022 as an example,[3] in the preparation stage, the NHSA released a working plan and guidelines, as well as convened expert committees for price negotiation. In the application stage, pharmaceutical companies would file for negotiation to get drugs reimbursed, based on which the NHSA would conduct an initial eligibility screening. The NHSA would then announce candidate drugs under consideration for reimbursement based on the screening results. It is important to note that since 2019, pharmaceutical companies have had the right to decide whether their drugs would participate in price negotiations.

At the review stage, expert committees assessed whether drugs were suitable for price negotiation or other pathways through joint reviews. For drugs eligible for price negotiation, experts determined drug specifications, reference drugs, and reimbursement conditions. At the negotiation stage, pharmaceutical companies submitted dossiers for evaluation by experts. These experts determined the drug price range

through budget impact analysis, cost-effectiveness analysis, and an insurance fund pressure test. Subsequently, the NHSA conducted price negotiations with pharmaceutical companies, and the agreed-upon price was established. During the results announcement stage, the NHSA released the updated version of the NRDL, which included drugs that had undergone successful price negotiations. Drugs listed in the NRDL were reimbursed by the NHSA.

References
1. Liu GG, Wu J, He X, Jiang Y. Policy Updates on Access to and Affordability of Innovative Medicines in China. Value Health Reg Issues. 2022; 30:59-66
2. Wen J, Li M, Jiang Y. Cost effectiveness of innovative anti-cancer drugs and reimbursement decisions in China. Health Policy Technol. 2023;12(2):100742
3. National Healthcare Security Administration (NHSA). Announcement on the release of the '2022 National Basic Medical Insurance, Work Injury Insurance, and Maternity Insurance Drug Catalog Adjustment Work Plan' and associated documents. 2022 [Accessed 2023 May 01]. http://www.nhsa.gov.cn/art/2022/6/29/art_109_8342.html.

Table S1. Outcomes of price negotiations in China from 2017 to 2022

Year of negotiation	Drugs to be negotiated	Drugs negotiated successfully			Anticancer drugs negotiated successfully		
		Total	New additions	Re-negotiations	Total	New additions	Re-negotiations
2017	44	36	36	0	15	15	0
2018	18	17	17	0	16	16	0
2019	150	97	70	27	18	8	9
2020	162	119	96	23	30	14	16
2021	117	94	67	27	38	18	20
2022	147	121	108	13	22	13	9

Note: Authors' analysis of data from the NHSA. We did not include traditional Chinese medicines for the treatment of cancer in this analysis.

Table S2. Overview of variables

Variables	Definition and explanation	Operationalization	Data Source
Dependent variables			
Reimbursement lag	Time from market authorization to reimbursement	Numeric	NMPA, NHSA

Reimbursement status	Whether the indication was listed in the NRDL by 2023	Categorical: 0 = not listed; 1 = listed	NHSA
Explanatory variables			
Launch price	Treatment price of indications over the expected treatment durations at launch	Numeric	The Drugdataexpy
Added survival	Added overall or progression-free survival life-months gained of indications supported by randomized controlled trials	Numeric	Publications of pivotal clinical trials
Quality of life	The comparable quality of life of the study drug in relation to its comparator of indications supported by randomized controlled trials	Categorical: 0 = reduction or not available, 1 = no difference, 2 = improvement	Publications of pivotal clinical trials
Overall response rate	Overall response rate of indications supported by single-arm clinical trials	Numeric	Publications of pivotal clinical trials
Control variables			
Drug type	The category of included indications specific to anticancer drugs	Categorical: 0 = small targeted molecule, 1 = targeted monoclonal antibody, 2 = immunotherapy, 3 = endocrine therapy & chemotherapy for indications supported by randomized controlled trials; 0 = small targeted molecule, 1 = targeted monoclonal antibody for indications supported by single-arm clinical trials	Drug labels and expertise
Cancer site	Disease area	Categorical: 0 = blood, 1 = lung, 2 = breast, 3 = other for therapeutic indications supported by randomized controlled trials; 0 = hematological, and 1= non- hematological for therapeutic indications supported by single-arm clinical trials	Drug labels
Domestic	Whether the drug was developed in China or imported from abroad	Categorical: 0 = imported, 1 = domestic.	Drug labels
Priority review	Market authorization pathway that expedites the review process for drugs	Categorical: 0 = non-priority review, 1 = priority review	CDE, NMPA
Conditional approval	Conditional marketing authorization for drugs	Categorical: 0 = non-conditional, 1= conditional	CDE, NMPA
Line of therapy	The order in which different therapies are given to people as their disease progresses	Categorical: 0 = other lines, 1 = first-line	Drug labels or clinical trials
Administration rote	Route of administration	Categorical: 0 = oral, 1= intravenous	Drug labels
Reference drug	The reference drug chose in randomized controlled trials of the study anticancer drug	Categorical: 0 = placebo, 1= active	Publications of pivotal clinical trials
Blind	The blind method used in the clinical trial	Categorical: 0 = no, 1= yes	Publications of pivotal clinical trials

Notes: CDE, NMPA, Center of Drug Evaluation, National Medical Products Administration; NHSA, National Healthcare Security Administration; NRDL, National Reimbursement Drug List.

Table S3. Information on 93 indications of anticancer drugs supported by randomized controlled trials in China.

No.	Generic name	Indication	Approval date	Reimbursement status	Reimbursement date	Reimbursement lag (days)	Treatment price at launch (CNY)	Survival (months)	Quality of life	Clinical trial number	Reference drug	Blind	Domestic	Administration route	Line of therapy	Priority review	Drug type	Cancer site
1	Lorlatinib	NSCLC	2022/4/27	reimbursed	2023/3/1	308	492,750	NA	improvement	NCT03052608	positive	no	no	oral	first-line	no	small targeted molecule	lung
2	Brigatinib	NSCLC	2022/3/24	reimbursed	2023/3/1	342	701,113	13	improvement	NCT02737501	positive	no	no	oral	first-line	no	small targeted molecule	lung
3	Rezvilutamide	prostate cancer	2022/3/24	reimbursed	2023/3/1	342	606,899	NA	improvement	NCT03520478	positive	no	yes	oral	first-line	no	endocrine therapy	prostate
4	Trastuzumab Emtansine	early stage breast cancer	2020/1/21	reimbursed	2023/3/1	1,135	656,796	NA	NA	NCT01772472	positive	no	no	intravenous	non-first-line	no	targeted monoclonal antibody	breast
5	Trastuzumab Emtansine	advanced breast cancer	2021/6/22	reimbursed	2023/3/1	617	516,054	4	NA	NCT00829166	positive	no	no	intravenous	non-first-line	no	targeted monoclonal antibody	breast
6	Ripretinib	gastrointestinal stromal tumor	2021/3/30	reimbursed	2023/3/1	701	628,729	8.5	improvement	NCT03353753	placebo	yes	no	oral	non-first-line	no	small targeted molecule	gastric
7	Utidelone	breast cancer	2021/3/11	reimbursed	2023/3/1	720	88,800	3.8	NA	NCT02253459.	positive	no	yes	intravenous	non-first-line	yes	chemotherapy	breast
8	Brentuximab	cutaneous T-cell lymphoma	2021/4/13	reimbursed	2023/3/1	687	572,000	13.2	no difference	NCT01578499	positive	no	no	intravenous	non-first-line	no	targeted monoclonal antibody	blood
9	Degarelix	prostate cancer	2018/9/11	reimbursed	2023/3/1	1,632	44,100	NA	NA	NA	positive	no	no	intravenous	first-line	no	endocrine therapy	prostate
10	Venetoclax	acute myeloid leukemia	2020/12/2	reimbursed	2023/3/1	819	331,075	5.1	no difference	NCT02993523	positive	yes	no	intravenous	first-line	yes	small targeted molecule	blood
11	Dalpiciclib	breast cancer	2021/12/31	reimbursed	2023/3/1	425	135,002	8.5	NA	NCT03927456	positive	yes	yes	oral	non-first-line	no	small targeted molecule	breast
12	Palbociclib	breast cancer	2018/7/31	reimbursed	2023/3/31	1,704	682,420	13.1	no difference	NCT01740427	positive	yes	no	intravenous	first-line	no	immunotherapy	breast
13	Sugemalimab	NSCLC	2021/12/20	not reimbursed	2023/12/31	741	247,500	5.1	NA	NCT03789604	positive	yes	yes	intravenous	first-line	no	immunotherapy	lung
14	Sugemalimab	NSCLC	2022/5/31	not reimbursed	2023/12/31	579	321,750	3.2	NA	NCT03728556	placebo	yes	yes	intravenous	non-first-line	no	immunotherapy	lung
15	Yttrium-90	metastatic colorectal cancer	2022/1/30	not reimbursed	2023/12/31	700	NA	0.5	no difference	NCT00724503	positive	yes	no	intravenous	first-line	no	radiotherapy	colorectal
16	Ramucirumab	gastric or gastroesophageal junction adenocarcinoma	2022/3/16	not reimbursed	2023/12/31	655	135,000	0.8	NA	NCT02898077	positive	yes	no	intravenous	non-first-line	no	targeted monoclonal antibody	gastric
17	Irinotecan	pancreatic adenocarcinoma	2022/4/12	not reimbursed	2023/12/31	628	NA	5.2	no difference	NCT01494506	positive	no	no	intravenous	non-first-line	no	chemotherapy	esophageal
18	Procarbazine	hodgkin's lymphoma	2022/4/19	not reimbursed	2023/12/31	621	19,723	NA	NA	NA	positive	no	no	oral	first-line	no	chemotherapy	blood
19	Afatinib	squamous cell carcinoma of the lung	2017/2/21	reimbursed	2018/9/30	586	39,758	1.1	improvement	NCT01523587	positive	no	no	oral	non-first-line	yes	small targeted molecule	lung
20	Afatinib	lung adenocarcinoma with EGFR mutations	2017/2/21	reimbursed	2018/9/30	586	130,775	-0.36	improvement	NCT01121393	positive	no	no	oral	first-line	yes	small targeted molecule	lung
21	Azacitidine	myelodysplastic syndromes	2017/4/24	reimbursed	2018/9/30	524	330,750	9.5	NA	NCT00071799	positive	no	no	intravenous	first-line	yes	chemotherapy	blood
22	Anlotinib	NSCLC	2018/5/8	reimbursed	2018/9/30	145	74,400	3.3	improvement	NCT02388919	placebo	yes	yes	oral	non-first-line	yes	small targeted molecule	lung
23	Osimertinib	NSCLC	2017/3/22	reimbursed	2018/9/30	557	728,640	4.3	improvement	NCT02151981	positive	no	no	oral	non-first-line	yes	small targeted molecule	lung

No.	Generic name	Indication	Approval date	Reimbursement status	Reimbursement date	Reimbursement lag (days)	Treatment price at launch (CNY)	Survival (months)	Quality of life	Clinical trial number	Reference drug	Blind	Domestic	Administration route	Line of therapy	Priority review	Drug type	Cancer site
24	Pazopanib	renal cell carcinoma	2017/2/21	reimbursed	2018/9/30	586	408,480	2.4	no difference	NCT00334282	placebo	yes	no	oral	first-line	no	small targeted molecule	renal
25	Regorafenib	metastatic colorectal cancer	2017/3/22	reimbursed	2018/9/30	557	97,199	2.5	no difference	NCT01584830	placebo	yes	no	oral	non-first-line	yes	small targeted molecule	colorectal
26	Regorafenib	gastrointestinal stromal tumor	2017/3/22	reimbursed	2018/9/30	557	185,483	3.9	no difference	NCT01271712	placebo	yes	no	oral	non-first-line	yes	small targeted molecule	gastric
27	Regorafenib	hepatocellular carcinoma	2017/12/5	reimbursed	2018/9/30	299	270,761	2.8	no difference	NCT01774344.	placebo	yes	no	oral	non-first-line	no	small targeted molecule	hepatobiliary
28	Ceritinib	NSCLC	2018/5/31	reimbursed	2018/9/30	122	476,589	-2	improvement	NCT01828112	positive	no	no	oral	non-first-line	yes	small targeted molecule	lung
29	Vemurafenib	melanoma	2017/3/10	reimbursed	2018/9/30	569	293,040	3.9	NA	NCT01006980	positive	no	no	oral	first-line	yes	small targeted molecule	skin
30	Ibrutinib	mantle-cell lymphoma	2017/8/24	reimbursed	2018/9/30	402	933,120	6.8	NA	NCT01646021	positive	no	no	oral	non-first-line	yes	small targeted molecule	blood
31	Ibrutinib	chronic lymphocytic leukemia or small lymphocytic lymphoma	2017/8/24	reimbursed	2018/9/30	402	1,992,600	2.6	improvement	NCT01578707	positive	no	no	oral	non-first-line	yes	small targeted molecule	blood
32	Ixazomib	multiple myeloma	2018/4/12	reimbursed	2018/9/30	171	496,800	2	no difference	NCT01564537	positive	yes	no	oral	non-first-line	yes	small targeted molecule	blood
33	Alectinib	NSCLC	2018/8/12	reimbursed	2020/1/1	507	752,378	23.9	NA	NCT02075840	positive	no	no	oral	first-line	yes	small targeted molecule	lung
34	Olaparib	ovarian cancer	2018/8/22	reimbursed	2020/1/1	497	1,545,839	12.9	no difference	NCT01874353	placebo	yes	no	oral	non-first-line	yes	small targeted molecule	ovarian
35	Pyrotinib	breast cancer	2018/8/12	reimbursed	2020/1/1	507	405,600	11.1	NA	NCT02422199	positive	no	yes	oral	non-first-line	yes	small targeted molecule	breast
36	Fruquintinib	metastatic colorectal cancer	2018/9/4	reimbursed	2020/1/1	484	81,252	2.7	NA	NCT02314819	placebo	yes	yes	oral	non-first-line	yes	small targeted molecule	colorectal
37	Ruxolitinib	myelofibrosis	2017/3/10	reimbursed	2020/1/1	1,027	627,850	NA	improvement	NCT00934544	placebo	no	no	intravenous	first-line	yes	small targeted molecule	blood
38	Pertuzumab	early HER2-positive breast cancer	2018/12/17	reimbursed	2020/1/1	380	357,200	NA	no difference	NCT01358877	positive	yes	no	intravenous	non-first-line	no	targeted monoclonal antibody	breast
39	Pertuzumab	early or locally advanced ERBB2-positive breast cancer	2019/8/16	reimbursed	2020/1/1	138	94,000	NA	NA	NCT02586025	positive	yes	no	intravenous	non-first-line	yes	targeted monoclonal antibody	breast
40	Dabrafenib	melanoma	2019/12/18	reimbursed	2021/3/1	439	414,720	8.2	improvement	NCT01597908	positive	no	no	oral	first-line	yes	small targeted molecule	skin
41	Dabrafenib	melanoma (adjuvant therapy)	2019/12/18	reimbursed	2021/3/1	439	456,192	NA	NA	NCT01682083	placebo	yes	no	oral	non-first-line	yes	small targeted molecule	skin
42	Enzalutamide	prostate cancer	2019/11/18	reimbursed	2021/3/1	469	1,307,573	10.7	improvement	NCT02003924	placebo	yes	no	oral	non-first-line	yes	endocrine therapy	prostate
43	Flumatinib	chronic myeloid leukemia	2019/11/22	reimbursed	2021/3/1	465	176,880	NA	NA	NCT02204644	positive	no	yes	oral	first-line	yes	small targeted molecule	blood
44	Camrelizumab	non-squamous NSCLC	2020/6/17	reimbursed	2021/3/1	257	217,800	7.39	NA	NCT03134872	positive	no	yes	intravenous	first-line	yes	immunotherapy	lung
45	Camrelizumab	oesophageal squamous cell carcinoma	2020/6/17	reimbursed	2021/3/1	257	118,800	2.1	improvement	NCT03099382	positive	no	yes	intravenous	non-first-line	yes	immunotherapy	gastric
46	Lenvatinib	hepatocellular carcinoma	2018/9/4	reimbursed	2021/3/1	909	287,280	1.3	no difference	NCT01761266	positive	no	no	oral	first-line	yes	small targeted molecule	hepatobiliary
47	Niraparib	ovarian cancer	2019/12/26	reimbursed	2021/3/1	431	1,574,370	15.5	no difference	NCT01847274	placebo	yes	yes	oral	non-first-line	yes	small targeted molecule	ovarian
48	Trametinib	melanoma (adjuvant therapy)	2019/12/18	reimbursed	2021/3/1	439	304,128	NA	NA	NCT01682083	placebo	yes	no	oral	non-first-line	yes	small targeted molecule	skin

No.	Generic name	Indication	Approval date	Reimbursement status	Reimbursement date	Reimbursement lag (days)	Treatment price at launch (CNY)	Survival (months)	Quality of life	Clinical trial number	Reference drug	Blind	Domestic	Administration route	Line of therapy	Priority review	Drug type	Cancer site
49	Trametinib	melanoma	2019/12/18	reimbursed	2021/3/1	439	552,960	6.4	improvement	NCT01584648	positive	yes	no	oral	first-line	yes	small targeted molecule	skin
50	Inetetamab	breast cancer	2020/6/17	reimbursed	2021/3/1	257	146,880	5.85	NA	NA	positive	no	yes	intravenous	non-first-line	yes	targeted monoclonal antibody	breast
51	Abemaciclib	advanced breast cancer （plus either anastrozole or letrozole）	2020/12/29	reimbursed	2022/1/1	368	588,998	13.42	no difference	NCT02246621	positive	yes	no	oral	first-line	yes	small targeted molecule	breast
52	Abemaciclib	advanced breast cancer (plus fulvestrant)	2020/12/29	reimbursed	2022/1/1	368	585,898	9.4	no difference	NCT02107703	positive	yes	no	oral	non-first-line	yes	small targeted molecule	breast
53	Apalutamide	prostate cancer	2020/8/12	reimbursed	2022/1/1	507	1,615,950	14	no difference	NCT01946204	placebo	yes	no	oral	non-first-line	yes	endocrine therapy	prostate
54	Apalutamide	prostate cancer	2020/8/12	reimbursed	2022/1/1	507	1,568,070	NA	no difference	NCT02489318.	positive	yes	no	oral	first-line	yes	endocrine therapy	prostate
55	Eribulin	breast cancer	2019/7/12	reimbursed	2022/1/1	904	119,400	0.9	NA	NCT02225470	positive	no	no	oral	non-first-line	no	chemotherapy	breast
56	Obinutuzumab	follicular lymphoma	2021/6/1	reimbursed	2022/1/1	214	172,000	NA	no difference	NCT01332968	positive	no	no	intravenous	first-line	yes	targeted monoclonal antibody	blood
57	Dacomitinib	NSCLC	2019/5/15	reimbursed	2022/1/1	962	87,166	7.1	reduction	NCT01774721	positive	no	no	oral	first-line	yes	small targeted molecule	lung
58	Daratumumab	multiple myeloma (plus lenalidomide and dexamethasone)	2021/4/27	reimbursed	2022/1/1	249	3,070,740	27	no difference	NCT02076009	positive	no	no	intravenous	non-first-line	yes	targeted monoclonal antibody	blood
59	Daratumumab	multiple myeloma (plus bortezomib and dexamethasone)	2021/4/27	reimbursed	2022/1/1	249	1,120,065	9.6	no difference	NCT02136134	positive	no	no	intravenous	non-first-line	yes	targeted monoclonal antibody	blood
60	Darolutamide	nonmetastatic, castration-resistant prostate cancer	2021/2/2	reimbursed	2022/1/1	333	349,286	22	improvement	NCT02200614	placebo	yes	no	oral	non-first-line	yes	endocrine therapy	prostate
61	Donafenib	hepatocellular carcinoma	2021/6/8	reimbursed	2022/1/1	207	90,926	1.8	NA	NCT02645981	positive	yes	yes	oral	first-line	yes	small targeted molecule	hepatobiliary
62	Fluzoparib	ovarian carcinoma	2021/6/22	reimbursed	2022/1/1	193	134,389	7.4	no difference	NCT03863860	placebo	yes	yes	oral	non-first-line	yes	small targeted molecule	ovarian
63	Niratinib	breast cancer	2020/4/27	reimbursed	2022/1/1	614	195,915	NA	reduction	NCT00878709	placebo	yes	no	oral	non-first-line	no	small targeted molecule	breast
64	Pomalidomide	multiple myeloma	2020/11/10	reimbursed	2022/1/1	417	67,200	4.6	improvement	NCT01311687	positive	no	yes	oral	non-first-line	yes	small targeted molecule	blood
65	Sulfatinib	extrapancreatic neuroendocrine tumors	2020/12/29	reimbursed	2022/1/1	368	129,321	5.4	no difference	NCT02588170.	placebo	yes	yes	oral	first-line	yes	small targeted molecule	other
66	Sulfatinib	pancreatic neuroendocrine tumors	2021/6/16	reimbursed	2022/1/1	199	139,035	7.2	no difference	NCT02589821	placebo	yes	yes	oral	non-first-line	no	small targeted molecule	other
67	Blinatumomab	acute lymphoblastic leukemia	2020/12/2	not reimbursed	2023/12/31	1,124	1,275,000	3.7	improvement	NCT02013167	positive	no	no	intravenous	first-line	yes	targeted monoclonal antibody	blood
68	Durvalumab	NSCLC	2019/12/6	not reimbursed	2023/12/31	1,486	169,078	2.4	NA	NCT03043872	positive	no	no	intravenous	first-line	no	immunotherapy	lung
69	Nivolumab	NSCLC	2018/6/15	not reimbursed	2023/12/31	2,025	148,000	3.2	NA	NCT01642004	positive	no	no	intravenous	non-first-line	no	immunotherapy	lung
70	Nivolumab	squamous-cell carcinoma of the head and neck	2019/9/29	not reimbursed	2023/12/31	1,554	74,000	2.4	improvement	NCT02105636	positive	no	no	intravenous	non-first-line	yes	immunotherapy	head and neck

No.	Generic name	Indication	Approval date	Reimbursement status	Reimbursement date	Reimbursement lag (days)	Treatment price at launch (CNY)	Survival (months)	Quality of life	Clinical trial number	Reference drug	Blind	Domestic	Administration route	Line of therapy	Priority review	Drug type	Cancer site
71	Nivolumab	gastric or gastro-oesophageal junction cancer	2020/3/11	not reimbursed	2023/12/31	1,390	74,000	1.12	NA	NCT02267343	placebo	yes	no	intravenous	non-first-line	yes	immunotherapy	gastric
72	Nivolumab	malignant pleural mesothelioma	2021/6/8	not reimbursed	2023/12/31	936	222,000	4	NA	NCT02899299	positive	no	no	intravenous	first-line	yes	immunotherapy	other
73	Nivolumab	advanced or recurrent oesophageal cancer	2021/8/25	not reimbursed	2023/12/31	858	277,044	2.2	no difference	NCT02872116	positive	no	no	intravenous	first-line	no	immunotherapy	gastric
74	Nivolumab	adjuvant therapy for esophageal cancer	2022/6/23	not reimbursed	2023/12/31	556	508,750	NA	no difference	NCT02743494	positive	yes	no	intravenous	non-first-line	no	immunotherapy	gastric
75	Nivolumab	esophageal squamous cell carcinoma	2022/6/23	not reimbursed	2023/12/31	556	277,044	2.5	no difference	NCT03143153	positive	no	no	intravenous	first-line	no	immunotherapy	gastric
76	Pembrolizumab	melanoma	2018/7/20	not reimbursed	2023/12/31	1,990	179,180	2.4	improvement	NCT01704287	positive	yes	no	intravenous	non-first-line	yes	immunotherapy	skin
77	Pembrolizumab	nonsquamous NSCLC	2019/3/28	not reimbursed	2023/12/31	1,739	501,704	11.3	improvement	NCT02578680	positive	yes	no	intravenous	first-line	yes	immunotherapy	lung
78	Pembrolizumab	squamous NSCLC	2019/11/22	not reimbursed	2023/12/31	1,500	358,360	5.5	improvement	NCT02775435	positive	yes	no	intravenous	first-line	yes	immunotherapy	lung
79	Pembrolizumab	locally advanced or metastatic NSCLC	2019/9/29	not reimbursed	2023/12/31	1,554	322,524	7.8	NA	NCT02220894	positive	no	no	intravenous	first-line	yes	immunotherapy	lung
80	Pembrolizumab	esophageal cancer	2020/6/17	not reimbursed	2023/12/31	1,292	215,016	3.6	no difference	NCT02564263	positive	no	no	intravenous	non-first-line	yes	immunotherapy	gastric
81	Pembrolizumab	squamous cell carcinoma of the head and neck	2020/12/8	not reimbursed	2023/12/31	1,118	179,180	4.2	no difference	NCT02358031	positive	no	no	intravenous	first-line	yes	immunotherapy	head and neck
82	Pembrolizumab	colorectal cancer	2021/6/8	not reimbursed	2023/12/31	936	573,376	8.3	improvement	NCT02563002	positive	no	no	intravenous	first-line	yes	immunotherapy	colorectal
83	Pembrolizumab	advanced oesophageal cancer	2021/9/1	not reimbursed	2023/12/31	851	394,196	2.6	no difference	NCT03189719	positive	yes	no	intravenous	first-line	yes	immunotherapy	gastric
84	Ipilimumab	malignant pleural mesothelioma	2021/6/8	not reimbursed	2023/12/31	936	112,000	4	NA	NCT02899299	positive	no	no	intravenous	first-line	yes	immunotherapy	other
85	Atezolizumab	hepatocellular carcinoma	2020/10/27	not reimbursed	2023/12/31	1,160	360,800	5.8	improvement	NCT03434379	positive	no	no	intravenous	first-line	yes	immunotherapy	hepatobiliary
86	Atezolizumab	small-cell lung cancer	2020/2/11	not reimbursed	2023/12/31	1,419	229,600	2	improvement	NCT02763579	positive	yes	no	intravenous	first-line	no	immunotherapy	lung
87	Atezolizumab	NSCLC	2021/4/27	not reimbursed	2023/12/31	978	262,400	7.1	NA	NCT02409342	positive	no	no	intravenous	first-line	yes	immunotherapy	lung
88	Atezolizumab	nonsquamous NSCLC	2021/6/22	not reimbursed	2023/12/31	922	295,200	4.5	NA	NCT02657434	positive	no	no	intravenous	first-line	no	immunotherapy	lung
89	Atezolizumab	II-IIIA NSCLC	2022/3/16	not reimbursed	2023/12/31	655	524,800	NA	NA	NCT02486718	placobo	no	no	intravenous	non-first-line	no	immunotherapy	lung
90	Gilteritinib	acute myeloid leukemia	2021/1/30	not reimbursed	2023/12/31	1,065	630,001	3.7	NA	NCT02421939	positive	no	no	oral	non-first-line	yes	small targeted molecule	blood
91	Radium-223 dichloride	prostate cancer	2020/8/26	not reimbursed	2023/12/31	1,222	NA	3.6	improvement	NCT00699751	placebo	yes	no	intravenous	non-first-line	yes	radiotherapy	prostate
92	LONSURF;Trifluridine and Tipiracil	colorectal cancer	2019/8/29	not reimbursed	2023/12/31	1,585	68,771	0.7	NA	NCT01955837	placebo	yes	no	intravenous	non-first-line	no	chemotherapy	colorectal
93	inotuzumab ozogamicin	acute lymphoblastic leukemia	2021/12/20	not reimbursed	2023/12/31	741	499,100	1.5	NA	NCT01564784	positive	no	no	intravenous	non-first-line	no	targeted monoclonal antibody	blood

Notes: NSCLC, non-small cell lung cancer. NA: not available. Reimbursement lag for unreimbursed drugs was calculated from the date of their regulatory approval to December 31, 2023. It's important to note that this lag may extend beyond the presented data, as these drugs may remain unreimbursed after the cutoff date.

Table S4. Information on 42 indications of anticancer drugs supported by single-arm clinical trials in China.

No.	Generic name	Indication	Approval date	Reimbursement status	Reimbursement date	Reimbursement lag	Treatment price at launch (CNY)	ORR	Clinical trial number	Domestic	Administration route	Line of therapy	Drug type	Conditional approval	Cancer site
1	Lorlatinib	NSCLC	2022/4/27	reimbursed	2023/3/1	308	506,250	48%	NCT01970865	no	oral	non-first-line	small targeted molecule	no	non-hematological
2	Brentuximab	Hodgkin's lymphoma	2020/5/12	reimbursed	2023/3/1	1,023	396,000	75%	NCT00848926	no	intravenous	non-first-line	targeted monoclonal antibody	no	hematological
3	Brentuximab	systemic anaplastic large-cell lymphoma	2020/5/12	reimbursed	2023/3/1	1,023	308,000	86%	NCT00866047	no	intravenous	non-first-line	targeted monoclonal antibody	no	hematological
4	Savolitinib	NSCLC	2021/6/22	reimbursed	2023/3/1	617	253,959	43%	NCT02897479	yes	oral	first - line	small targeted molecule	yes	non-hematological
5	Olverembatinib	chronic myeloid leukemia in chronic phase	2021/11/24	reimbursed	2023/3/1	462	487,500	NA	NCT03883087	yes	oral	non-first-line	small targeted molecule	yes	hematological
6	Olverembatinib	chronic myeloid leukemia in accelerated phase	2021/11/24	reimbursed	2023/3/1	462	438,750	NA	NCT03883100	yes	oral	non-first-line	small targeted molecule	yes	hematological
7	Carfilzomib	multiple myeloma	2021/7/9	reimbursed	2023/3/1	600	243,542	36%	NCT03029234	no	intravenous	non-first-line	small targeted molecule	yes	hematological
8	Pralatrexate	peripheral T-Cell lymphoma	2020/8/26	not reimbursed	2023/12/31	1,222	NA	52%	NCT03349333	no	intravenous	non-first-line	small targeted molecule	yes	hematological
9	Avapritinib	gastrointestinal stromal tumor	2021/3/30	not reimbursed	2023/12/31	1,006	NA	88%	NCT02508532	no	oral	first - line	small targeted molecule	yes	non-hematological
10	Axicabtagene ciloleuce	large B-cell lymphoma	2021/6/22	not reimbursed	2023/12/31	922	1,200,000	79%	FKC876-2018-001	yes	intravenous	non-first-line	small targeted molecule	no	hematological
11	Pralsetinib	NSCLC	2021/3/23	not reimbursed	2023/12/31	1,013	486,000	57%	NCT03037385	no	oral	non-first-line	small targeted molecule	yes	non-hematological
12	Pralsetinib	thyroid cancer	2022/3/8	not reimbursed	2023/12/31	663	486,000	89%	NCT03037385	no	oral	non-first-line	small targeted molecule	yes	non-hematological
13	Ivosidenib	leukemia	2022/1/30	not reimbursed	2023/12/31	700	286,179	42%	NCT02074839	no	oral	first - line	small targeted molecule	yes	hematological
14	Relmacabtagene	large B-cell lymphoma	2021/9/1	not reimbursed	2023/12/31	851	1,290,000	60%	NCT04089215	yes	intravenous	first - line	small targeted molecule	yes	hematological
15	Envafolimab	solid tumor	2021/11/24	not reimbursed	2023/12/31	767	215,280	39%	CTR20181127	yes	intravenous	non-first-line	targeted monoclonal antibody	yes	non-hematological
16	Larotrectinib	solid tumor	2022/4/8	not reimbursed	2023/12/31	632	NA	67%	NCT02122913<br>NCT02637687<br>NCT02576431	no	oral	non-first-line	small targeted molecule	no	non-hematological
17	Sintilimab	relapsed or refractory classical Hodgkin's lymphoma	2018/12/24	reimbursed	2020/1/1	373	188,112	80%	NCT03114683	yes	intravenous	non-first-line	targeted monoclonal antibody	yes	hematological
18	Almonertinib	EGFR T790M advanced or metastatic NSCLC	2020/3/17	reimbursed	2021/3/1	349	723,240	69%	NCT02981108	yes	oral	first - line	small targeted molecule	yes	non-hematological
19	Camrelizumab	relapsed or refractory classical Hodgkin's lymphoma	2019/5/29	reimbursed	2021/3/1	642	534,600	77%	NCT03155425	yes	intravenous	non-first-line	targeted monoclonal antibody	yes	hematological
20	Toripalimab	advanced or metastatic Melanoma	2018/12/17	reimbursed	2021/3/1	805	72,000	17%	NCT03013101	yes	intravenous	non-first-line	targeted monoclonal antibody	yes	non-hematological
21	Tislelizumab	relapsed or refractory classical Hodgkin's lymphoma	2019/12/26	reimbursed	2021/3/1	431	277,888	77%	NCT03209973	no	intravenous	first - line	targeted monoclonal antibody	yes	hematological

No.	Generic name	Indication	Approval date	Reimbursement status	Reimbursement date	Reimbursement lag	Treatment price at launch (CNY)	ORR	Clinical trial number	Domestic	Administration route	Line of therapy	Drug type	Conditional approval	Cancer site
22	Tislelizumab	advanced or metastatic urothelial bladder cancer	2020/4/9	reimbursed	2021/3/1	326	106,880	25%	NCT04004221	no	intravenous	non-first-line	targeted monoclonal antibody	yes	non-hematological
23	Zanubrutinib	mantle cell lymphoma	2020/6/2	reimbursed	2021/3/1	272	584,777	84%	NCT03206970	yes	oral	first - line	small targeted molecule	yes	hematological
24	Zanubrutinib	chronic lymphocytic leukemia/small lymphocytic lymphoma	2020/6/2	reimbursed	2021/3/1	272	296,626	85%	NCT03206918	yes	oral	non-first-line	small targeted molecule	yes	hematological
25	Orelabrutinib	mantle cell lymphoma	2020/12/25	reimbursed	2022/1/1	372	162,992	78%	NCT03494179	yes	oral	non-first-line	small targeted molecule	yes	hematological
26	Orelabrutinib	chronic lymphocytic leukemia/ small lymphocytic lymphoma	2020/12/25	reimbursed	2022/1/1	372	161,912	74%	NCT03493217	yes	oral	non-first-line	small targeted molecule	yes	hematological
27	Daratumumab	multiple myeloma	2019/7/4	reimbursed	2022/1/1	912	604,080	29%	NCT01985126	no	intravenous	non-first-line	targeted monoclonal antibody	yes	hematological
28	Ensartinib	NSCLC	2020/11/7	reimbursed	2022/1/1	420	388,886	52%	NCT03215693	yes	oral	non-first-line	small targeted molecule	yes	non-hematological
29	Furmonertinib	EGFR T790M advanced or metastatic NSCLC	2021/3/2	reimbursed	2022/1/1	305	332,572	74%	NCT03452592	yes	oral	non-first-line	small targeted molecule	yes	non-hematological
30	Fluzoparib	ovarian cancer	2020/12/11	reimbursed	2022/1/1	386	182,950	70%	NCT03509636	yes	oral	first - line	small targeted molecule	yes	non-hematological
31	Pamiparib	advanced high-grade ovarian cancer	2021/4/30	reimbursed	2022/1/1	246	173,250	68%	NCT03333915	yes	oral	non-first-line	small targeted molecule	yes	non-hematological
32	Disitamab vedotin	gastric cancer	2021/6/8	reimbursed	2022/1/1	207	364,500	24%	NCT03556345	yes	intravenous	non-first-line	targeted monoclonal antibody	yes	non-hematological
33	Cadonilimab	cervical cancer	2022/6/28	not reimbursed	2023/12/31	551	317,280	31%	NCT04380805	yes	intravenous	non-first-line	targeted monoclonal antibody	yes	non-hematological
34	Serplulimab	solid tumor	2022/3/22	not reimbursed	2023/12/31	649	167,640	31%	NCT03941574	yes	intravenous	non-first-line	targeted monoclonal antibody	yes	non-hematological
35	Duvelisib	lymphoma	2022/3/16	not reimbursed	2023/12/31	655	106,500	42%	NCT01882803	no	oral	non-first-line	small targeted molecule	yes	hematological
36	Pemigatinib	cholangiocarcinoma	2022/3/29	not reimbursed	2023/12/31	642	665,470	50%	NCT04256980	no	oral	non-first-line	small targeted molecule	no	non-hematological
37	Dinutuximab beta	neuroblastoma	2021/8/12	not reimbursed	2023/12/31	871	2,383,920	NA	NCT01704716	no	intravenous	non-first-line	targeted monoclonal antibody	no	non-hematological
38	Iodine[131I] metuximab	hepatic carcinoma	2021/4/30	not reimbursed	2023/12/31	975	NA	8%	NA	yes	intravenous	non-first-line	targeted monoclonal antibody	no	non-hematological
39	Penpulimab	classical Hodgkin lymphoma	2021/8/3	not reimbursed	2023/12/31	880	273,000	89%	NCT03722147	yes	intravenous	non-first-line	targeted monoclonal antibody	yes	hematological
40	Selinexor	multiple myeloma	2021/12/4	not reimbursed	2023/12/31	747	134,610	25%	NCT02336815	no	oral	non-first-line	small targeted molecule	yes	hematological
41	Zimberelimab	classical Hodgkin lymphoma	2021/8/25	not reimbursed	2023/12/31	858	NA	91%	NCT03655483	yes	intravenous	non-first-line	targeted monoclonal antibody	yes	hematological
42	Sacituzumab govitecan-hzi	breast cancer	2022/6/10	not reimbursed	2023/12/31	569	284,000	38.80%	NCT04454437	no	intravenous	non-first-line	targeted monoclonal antibody	yes	non-hematological

Notes: ORR, overall response rate. NSCLC, non-small cell lung cancer. NA: not available. Reimbursement lag for unreimbursed drugs was calculated from the date of their regulatory approval to December 31, 2023. It's important to note that this lag may extend beyond the presented data, as these drugs may remain unreimbursed after the cutoff date.
